# 3D Structure Prediction of Human β1-Adrenergic Receptor via Threading-Based Homology Modeling for Implications in Structure-Based Drug Designing

**DOI:** 10.1371/journal.pone.0122223

**Published:** 2015-04-10

**Authors:** Zaheer Ul-Haq, Maria Saeed, Sobia Ahsan Halim, Waqasuddin Khan

**Affiliations:** 1 Dr. Panjwani Center for Molecular Medicine and Drug Research, International Center for Chemical and Biological Sciences, University of Karachi, Karachi, Pakistan; 2 National Centre of Excellence in Molecular Biology, University of the Punjab, Lahore, Pakistan; MRC National Institute for Medical Research, UNITED KINGDOM

## Abstract

Dilated cardiomyopathy is a disease of left ventricular dysfunction accompanied by impairment of the β_1_-adrenergic receptor (β_1_-AR) signal cascade. The disturbed β_1_-AR function may be based on an elevated sympathetic tone observed in patients with heart failure. Prolonged adrenergic stimulation may induce metabolic and electrophysiological disturbances in the myocardium, resulting in tachyarrhythmia that leads to the development of heart failure in human and sudden death. Hence, β_1_-AR is considered as a promising drug target but attempts to develop effective and specific drug against this tempting pharmaceutical target is slowed down due to the lack of 3D structure of *Homo sapiens* β_1_-AR (*hs*βADR1). This study encompasses elucidation of 3D structural and physicochemical properties of *hs*βADR1 *via* threading-based homology modeling. Furthermore, the docking performance of several docking programs including Surflex-Dock, FRED, and GOLD were validated by re-docking and cross-docking experiments. GOLD and Surflex-Dock performed best in re-docking and cross docking experiments, respectively. Consequently, Surflex-Dock was used to predict the binding modes of four *hs*βADR1 agonists. This study provides clear understanding of *hs*βADR1 structure and its binding mechanism, thus help in providing the remedial solutions of cardiovascular, effective treatment of asthma and other diseases caused by malfunctioning of the target protein.

## Introduction

G-protein coupled receptor (GPCR) superfamily constitutes the largest family of receptors in cell responsible for mediating the effects of over 50% of drugs in the market now-a-days [1–8]. GPCRs are involved in the transmission of a variety of signals to the interior of the cell and can be activated by a diverse range of small molecules including nucleotides, amino acids, peptides, proteins and odorants. Activation of GPCRs results in a conformational change followed by a signal cascade that passes information to the inside of the cell by interacting with a protein known as heterotrimeric G-proteins. There are three main classes of GPCRs (A, B and C) depending on their sequence similarity to Rhodopsin (Rho) (Class A). Class A GPCRs is the largest group and encompasses a wide range of receptors including receptors for odorants, adenosine, β-adrenergic and Rhodopsin [1–8]. The β-adrenergic receptors (β-ARs) are G_s_ protein–coupled receptors that play important roles in cardiovascular function and disease, through serving as receptors for the neurohormones: norepinephrine and epinephrine. Norepinephrine released from cardiac sympathetic nerves activates myocyte β_1_-ARs, which activates adenylyl cyclase via stimulatory G-protein (G_s_). The rise in the intracellular [cAMP] level causes the phosphorylation of several intracellular proteins by means of cAMP-dependent protein kinase A. Such type of activated β_1_-AR results in an increased cardiac inotropy, lusitropy, and chronotropy and the secretion of rennin, all of which contribute to regulate the cardiac functions and blood pressure [9–10]. β_1_-AR predominates in the heart, representing about 80% of the myocardial β-ARs; thus, they tend to be viewed as the most significant β-ARs with respect to the cardiovascular system. β_1_- and β_2_-ARs in kidneys stimulate the release of renin, thereby playing a role in the activation of renin-angiotensin-aldosterone system [9–10].

The role of β-ARs in cardiovascular function and disease is also highlighted by the significant roles of drugs whose actions are based on binding to the β-ARs blockers (β-blockers). β-blockers represent first line therapy for the management of chronic heart failure, hypertension, acute and post myocardial infarction patients, chronic stable angina, and unstable angina [11]. They are also commonly used to control the symptoms of atrial fibrillation and other arrhythmias [11]. There are no cardiovascular drugs that have a wider range of indications than β-blockers, making them a critical drug class for the management of cardiovascular disease. The availability of uses for β-blockers also suggests that the activation of β-ARs, or the sympathetic nervous system (SNS), plays an essential function in most cardiovascular diseases. The fact that β_1_-AR selective antagonists are equivalent to non-selective blockers in essentially all situations provides additional evidence that β_1_-ARs are the more important β-receptors with respect to cardiovascular disease. The development of a large number of rational inhibitors that have the ability to modulate the activity of such receptors has been a major goal for the pharmaceutical industries to improve the clinical treatment of various disease including hypertension, heart failure and asthma [12]. However, finding specific drug against a particular β-ARs drug target is a slow and laborious process. Furthermore, the lack of 3D structure of *hs*ADRB1 is an obstacle in the identification of specific drug like molecules.

On the other hand, the development of computational approaches for drug designing can be effectively carried out with low cost [13–14]. The use of computational techniques in drug discovery and development process is rapidly gaining popularity, implementation and appreciation. There will be an intensifying effort to apply computational power to combine biological and chemical space in order to rationalize the drug discovery, designing and optimization phenomena. Today, Computer Aided Drug Design (CADD) is based on the knowledge of structure, either of the receptor, or that of the ligand. The former is described as Structure-based while the later as Ligand-based drug designing. Because it is difficult and time-consuming to obtain experimental structures from methods such as X-ray crystallography and protein NMR for every protein of interest, homology modeling is a widely used *in silico* technique providing the useful structural models for generating hypotheses about a protein's function and directing further experimental work [15].

The main objective of this study is to employ “*in silico”* homology modeling technique to construct the 3D structure of *hs*ADRB1 that will be used to identify and characterize new inhibitors for *hs*ADRB1 by structure-based computational approaches. This model serves as a starting point to gain knowledge of protein-ligand interactions and the structural requirements of active site of protein.

## Material and Methods

### Computational resources and tools

**Fig 1:**
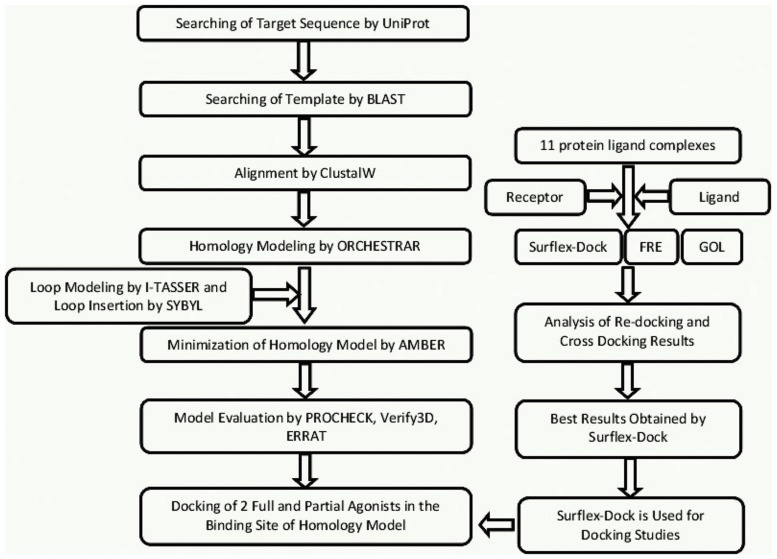
Schematics of strategy implemented towards successful homology modeling of *hs*βADR1 and its docking studies.

Computational studies were performed on Intel Xeon Quad core (2.33 GHz processor) server installed with LINUX OS (openSUSE Version 12.0). Multiple sequence alignment was carried out by ClustalW of the closest homologue identified by NCBI p-Blast to find out the identity, similarity and gap region between the target and template [16]. Homology modeling was accomplished by ORCHESTRAR [17] implemented in BioPolymer module of SYBYL7.3 [18]. An online server, I-TASSER [19], was used for modeling a region absent in template structure. The finally selected model of *hs*βADR1was minimized by AMBER (Version10.0) [20]. Stereochemical properties of modeled protein structure were validated by PROCHECK [21], Verify3D [22] and ERRAT [23]. Molecular docking experiments were conducted by Surflex-Dock implemented in SYBYL (Version 7.3) [24], FRED (Version 2.2.5) [25] and GOLD (Version 2.5) [26]. UCSF CHIMERA [27–28] and MOE [29] were used for visualization purpose. The flowchart of work plan is illustrated in (Fig 1).

### Searching of template sequences and multiple sequence alignment

The sequence of *hs*βADR1 (AC No: P08588) was retrieved from UniProt KB [30]. This target sequence comprises of 477 amino acid residues was submitted to NCBI-Protein BLAST [31] to search the closest homologue. Top-ranked template sequences as determined by BLAST were subjected for multiple sequence alignment on the basis of optimized E-value of the specified target sequence (Table 1). However, *Meleagris gallopavo* β_1_-AR (*Mg*βADR1, **PDB ID: 2Y00**) retrieved as the closest homologue, was manually edited for optimal alignment along with the target sequence. Best alignment was selected based on alignment score and the reciprocal position of the conserved amino acid residues across the members of class A GPCR superfamily.

**Table 1:** Top-ranked template sequences obtained by BLAST results.

Ranking No.	PDB ID	LIG ID*	Total Score	Query Coverage	Max. Identity	Positives	e-Value
**1**	2Y00	Y00	473	73%	68%	75%	3×10^-165^
**2**	2VT4	P32	466	73%	68%	75%	1×10^-162^
**3**	2R4R	N/A	412	79%	54%	66%	1×10^-140^
**4**	3KJ6	N/A	412	79%	54%	66%	1×10^-140^
**5**	2R4S	N/A	408	74%	56%	66%	3×10^-139^
**6**	3SN6	P0G	410	74%	56%	66%	1×10^-137^
**7**	4GBR	CAU	291	43%	67%	81%	4×10^-94^
**8**	3P0G	P0G	418	79%	54%	75%	5×10^-92^
**9**	2RH1	CAU	418	79%	54%	76%	5×10^-92^
**10**	3PDS	ERC	410	72%	60%	78%	1×10^-90^

*Y00 = Dobutamine, P32 = Cyanopindolol, P0G = Nanobody (Nb35), CAU = Carazolol, ERC = FAUC50. RET = Retinal, P32 = Cyanopindolol, CAU = Carazolol, Y00 = Dobutamine, WHJ = Carmoterol, 5FW = Isoprenaline, 68H = Salbutamol, TIM = Timolol, JRZ = ICI 118,551.

### Numbering scheme for GPCRs

The confined Ballesteros and Weinstein numbering scheme [32] was used to identify the transmembrane (TM) segments relative to the conserved position of amino acids in TM helices assigned as locant.50 shareing the common features in all class A GPCR superfamily. This is followed by the representation of amino acids TM helix numbers. The immediately preceding and following the .50 residue are numbered .49 and .51, respectively.

### Homology modeling of *hs*βADR1

ORCHESTRAR is specifically designed for homology or comparative protein modeling that identifies structurally conserved regions (SCRs), models loops using model-based and *ab-initio* methods, models side chains, and combine them all to prepare a final model. Initially, a homology model was generated by ORCHESTRAR that lacks a region of 45 amino acid residues (209–254) of the cytoplasmic loop of TM5 located within the target sequence but absent in the template structure. This region was modeled by I-TASSER, an integrated platform for automated protein structure and function whose prediction is based on sequence-to-structure-to-function paradigm as per multiple threading alignments by LOMETS [33]. The model generated by I-TASSER was named as sub-model 1. Five sub-models were evaluated by replica-exchange Monte Carlo simulations with low free-energy states, spatial restrains and alignments TM regions [34] to identify the best structural alignment almost closed to the structural analogs on the basis of structural similarity. Any further steric clashes were removed to refine the coordinates, and the final results of all sub-models were based on sequence-structure-function paradigm obtained from the consensus of structural similarity and confidence score (C-score) of I-TASSER server. C-score value is the quality for the predicted sub-model on the basis of threading method. Stereochemical properties of each sub-model were evaluated and the best selected sub-model was incorporated to the homology model of *hs*βADR1, generated previously by ORCHESTRAR and after insertion of the model the finalized modelled is subjected for optimization.

### Structure optimization of homology model of *hs*βADR1

Homology model of *hs*βADR1 generated by ORCHESTRAR was minimized by SYBYL using conjugate gradient and steepest descent method with 10,000 iterations each. The selected sub-model generated by I-TASSER was also individually minimized to 10,000 cycles by AMBER10, followed by the insertion of sub-model into the homology model of *hs*βADR1 by chain joining option in SYBYL. The finally generated model is minimized further to 30,000 cycles using ff99SB force field by AMBER10.

### Molecular Docking

#### Selection of complexes for re-docking and cross-docking validation

To identify a suitable docking program for the docking of *hs*βADR1 agonists, re-docking and cross-docking experiments were performed by Surflex-Dock, FRED, and GOLD. Six βADR1-ligand complexes, three βADR2-ligand complexes and two Rhodopsin-ligand complexes were retrieved from PDB. The details of the protein-ligand complexes used in this study are summarized in Table 2 and S1 Fig. Selection of complexes was based on following criteria: availability of the protein-ligand complexes, the crystallographic resolution of protein-ligand complexes should be ≤3 Ǻ, the binding interaction of the protein-ligand complexes should be known. Cross-docking experiments conducted in using multiple docking methods with their scoring function are utilized in this study mentioned in S2, S3 and S4 Tables. The details of docking methodology are discussed in supporting informations.

**Table 2:** GPCR complexes utilized in re-docking and cross-docking setup.

S. No.	PDB ID	Resolution (Å)	Source	LIG ID*	No. of Rotatable Bonds	pEC_50_ Value (μM)	References
**1**	1GZM	2.65Å	*Bos Taurus* (Rhodopsin)	RET	4	—	[52]
**2**	1HZX	2.8 Å	*Bos Taurus* (Rhodopsin)	RET	4	—	[53]
**3**	2VT4	2.7 Å	*Meleagris gallopovo* βADR1	P32	7	-9.72±0.09	[ 38]
**4**	2RH1	2.4 Å	*Homo sapiens/Enterobacteria phageT4* βADR2	CAU	6	-11.3±1.2	[54]
**5**	2Y00	2.5 Å	*Meleagris gallopovo* βADR1	Y00	7	-6.24±0.04	[35]
**6**	2Y01	2.6 Å	*Meleagris gallopovo* βADR1	Y00	7	-6.24±0.04	[35]
**7**	2Y02	2.6 Å	*Meleagris gallopovo* βADR1	WHJ	7	-8.37±0.07	[35]
**8**	2Y03	2.85 Å	Meleagris gallopovo βADR1	5FW	4	-7.86±0.10	[35]
**9**	2Y04	3.05 Å	Meleagris gallopovo βADR1	68H	5	-5.25±0.04	[35]
**10**	3D4S	2.8 Å	*Homo sapiens* βADR2	TIM	7	-5.55±0.14	[55]
**11**	3NY8	2.84 Å	*Homo sapiens/Enterobacteria phage T4* βADR2	JRZ	6	-9.08±0.18	[56]

*RET = Retinal, P32 = Cyanopindolol, CAU = Carazolol, Y00 = Dobutamine, WHJ = Carmoterol, 5FW = Isoprenaline, 68H = Salbutamol, *TIM = Timolol, JRZ = ICI 118,551

### RMSDs and rankings

The re-docking results were analyzed to check the ability of docking programs to correctly identify the bound conformation of co-crystallized ligand in the top-ranked solution. RMSDs were calculated between the corresponding co-crystallized ligand against its predicted docked pose. Cross-docking experiments were conducted to identify which docking program exactly identified its cognate ligand among the diverse set of ligands within the top-ranked solution. For cross-docking, 11 complexes were extracted from PDB in which eight proteins are homodimers (chain A and chain B) while the rest of three are monomers (chain A). For those proteins that are present as homodimers, ligands were docked into both chains. Overall, 19 complexes were evaluated for cross-docking. The results were quantified as Best (1–3 position), Moderate (4–5 position) and Worst when the cognate ligand ranks position lowers than 5 within its cognate protein, respectively.

## Results and Discussion

### BLAST results and multiple sequence alignments

BLAST predicted *Mg*βADR1 (PDB ID: 2Y00) [35] as the best match for *hs*βADR1 with 68% identity and 75% positivity (with an e-value of 3×10^-165^). 2R4R, 3KJ6, 3P0G and 2RH1 have 79% while 2R4S and 3SN6 have 74% query coverage, more sequence coverage than observed for 2Y00 (73%). Since 2R4R, 3KJ6 and 24RS are available as apo form with fewer scores, identity and positive values, these structures were not used in this study. Similarly, the complexes 3P0G, 2RH1 and 3SN6 were not used for the modeling of *hs*βADR1 structure due to their lower scores. Hence, 2Y00 is used according to the BLAST results but to establish more confidence on the top-ranked search, we opted for two sorts of multiple sequence alignments: raw multiple sequence alignment and manually-edited multiple sequence alignment. For raw alignment, the ten top-ranked templates sequences (**2Y00, 2VT4, 2R4R, 3KJ6, 2R4S**, **3SN6, 4GBR, 3P0G, 2RH1 and 3PDS**) were aligned against the target sequence illustrated in S2 Fig and S1 Table. For manually-edited alignment, both the target and template (**2Y00**) sequences were truncated. The first 50 residues from N-terminus and 84 residues (393–477) from C-terminus were omitted from the target sequence due to the absence of corresponding homologous sequence in the template and has no important residue which is necessary to be in helical segments. The template sequence has 483 amino acid residues whereas the structure of 33–368 residues has been resolved (total 315 residues as some residues are missing). The first 3 residues (33–36) from N-terminus and 14 residues (337–351) from C-terminus were omitted. Finally, 342 residues of target sequence was aligned with ten top-ranked BLAST search, 2Y00 (297 residues), 2VT4, 2R4R, 3KJ6, 2R4S, 3SN6, 4GBR, 3P0G, 2RH1 and 3PDS The average alignment score for manually edited multiple sequence alignment is better (76.47) than the score obtained by raw multiple sequence alignment (74.89). Overall, there are 15 instances where alignments are improved, 7 alignments are improved when the target sequence is aligned with the rest of the sequences and 8 times the alignments have better quality when the template sequence is aligned with the remaining sequences.

**Fig 2:**
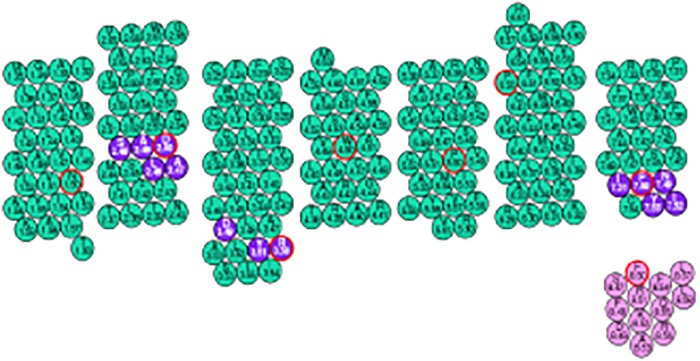
Snake plot representation of 7-TM helix regions of *hs*βADR1. TM helices depicted in green circles (black outline). Green circles with red outline, Blue circles with black outline and Purple color circles represents the conserved residues for all class A GPCRs, conserved motifs of class A GPCRs and helix 8 implemented in 7-TM helices of *hs*βADR1, respectively.

The generalized Ballesteros and Weinstein numbering scheme is beneficial for the understanding, recognition and structural alignments of GPCRs family [32]. The Ballesteros and Weinstein numbering is illustrated in (Fig 2) and the conserved amino acid residues of class A GPCRs is tabulated in Table 3. Ballesteros and Weinstein numbering is useful for the understanding of integrating methods for the construction of 3D models and computational probing of structure-function relations in GPCRs. These criteria pertain to the selection of correct inputs for the a1ignment programs and to structural considerations applicable to checking and refining the sequence alignments generated by alignment programs. This selection criterion depends on the information that is determined by the extent of homology among the compared sequences. Alignment of sequences with intermediate homologies (i.e., 30–70%) can identify continuous patterns of conservation distributed over the entire sequence. Such patterns provide structural inferences based on conservation.

**Table 3:** Identification and comparison of conserved residues of class A GPCRs located within TM helices in template *Mg*βADR1 and modeled *hs*βADR1.

TM Helices	Conserved Residues of Class A GPCRs	Conserved Identifier of Class A GPCRs	Amino Acid Positions in *Mg*βADR1	Amino Acid Positions in *hs*βADR1	Amino Acid Identifiers in *Mg*βADR1	Amino Acid Identifiers in *hs*βADR1
**TM 1**	Asn	N1.50	29	26	N1.50(29)	N1.50(26)
**TM 2**	Asp	D2.50	57	54	D2.50(57)	D2.50(54)
**TM 3**	Arg	R3.50	109	106	R3.50(109)	R3.50(106)
**TM 4**	Trp	W4.50	136	133	W4.50(136)	W4.50(133)
**TM 5**	Pro	P5.50	189	186	P5.50(189)	P5.50(186)
**TM 6**	Pro	P6.50	247	289	P6.50(247)	P6.50(289)
**TM 7—Helix 8**	Pro, Phe	P7.50, F8.50	282,289	324, 333	P7.50(282), F8.50(289)	P7.50(324), F8.50(333)

### Generation of the homology model of *hs*βADR1

**Fig 3:**
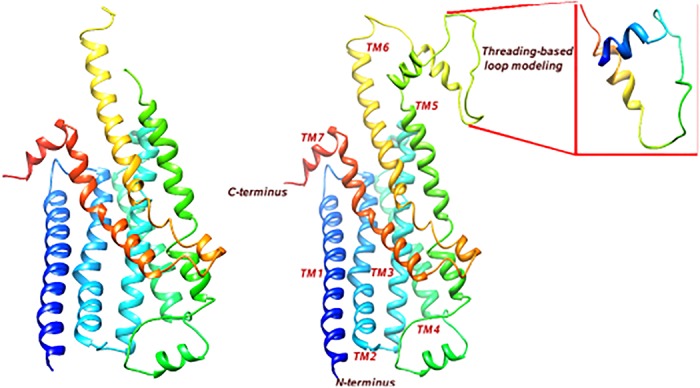
3D presentation of *hs*βADR1 homology model generated by ORCHESTRAR (A) without sub-model region, and (B) with sub-model region (amino acid residues from Val209 to Lys254) obtained by I-TASSER.

The *hs*βADR1 model is selected after structural comparison, superimposition and PROCHECK results (Fig 3A). ORCHESTRAR generated homology model using template 2Y00 was incomplete since the structure of residues 209–254 was missing. ORCHESTRAR fills the gap but not more than1–12 residues long, therefore, an *ab-initio* based threading method is used to predict the structure of missing region (S3 Fig). Subsequently, five sub-models were generated. Each sub-model is further analyzed by Ramachandran plot (Table 4). Among them, sub-model 1 is selected on the basis of highest C-score (-2.43) and stereochemical properties. The C-score value being lower than -1.5 likely indicates a lack of an appropriate template within the I-TASSER library. The selected sub-model 1 was subsequently inserted into the homology model of *hs*βADR1 by SYBYL. The C-terminus Val208 and the N-terminus Lys254 of the homology model is connected with the N-terminus Val209 and the C-terminus Thr255 of sub-model 1, respectively (Fig 3B).

**Table 4:** Evaluations of sub-models generated by I-TASSER.

Sub-models Generated by I-TASSER	Confidence Score (C-score)	PROCHECK Results for I-TASSER Generated Sub-Models			
		Residues in Additionally Allowed Region	Residues in Additionally Allowed Region	Residues in Generously Allowed Region	Residues in Generously disallowed Region
**Sub-model 1**	-2.43	16 aa (60%)	7 aa (28%)	1 aa (4%)	1 aa (4%)
**Sub-model 2**	-3.53	19 aa (76%)	3 aa (12%)	2 aa (8%)	1 aa (4%)
**Sub-model 3**	-4.05	14 aa (56%)	11aa (44%)	0 aa (0.0%)	0 aa (0.0%)
**Sub-model 4**	-4.46	14 aa (56%)	8 aa (32%)	2 aa (8%)	1 aa (4.0%)
**Sub-model 5**	-4.72	13 aa (52%)	8 aa (32%)	1 aa (4%)	3 aa (12%)

### Validation of *hs*βADR1 homology model

**Fig 4:**
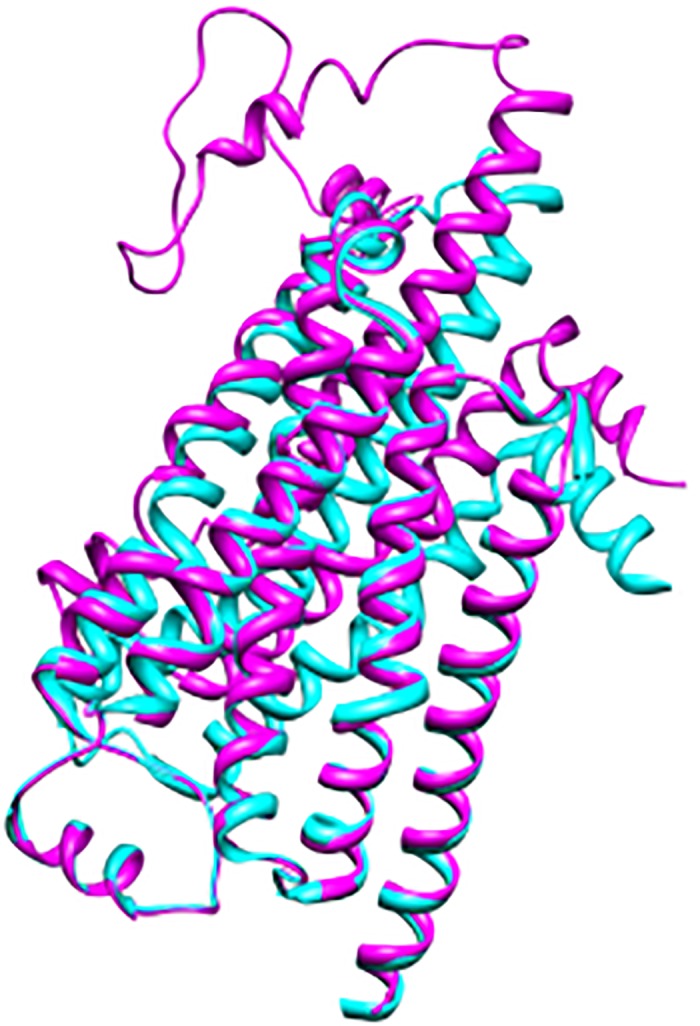
Superimposed structure of template (cyan colored) and homology model of hsβADR1 (magenta colored).

Several approaches were adopted to evaluate the geometrical and structural consistency of the homology model of *hs*βADR1. The structural and physicochemical properties of the model were validated by PROCHECK. The Ramachandran plot generated by PROCHECK is depicted in S4 Fig. The Ramachandran plot reveals that the model has a good geometrical consistency. According to the Ramachandran plot, ~85%, 13.5% and 1.3% residues are located within the favorable, allowed and the generously allowed regions, respectively while only one residue (Ile208) is found to be in the disallowed region. The visual inspection revealed that Ile208 is far away from the active site region and do not lie within 5Å of active site. Additionally, stereochemical properties of the model were validated by Verify3D web server. Verify3D evaluated the local environment and inter-residue contacts in the model. Ideally, the 3D-1D profile for each of the 20 amino acids should be in range of 0–0.2. Values less than zero are considered as inaccurate for the homology model. The Verify3D plot of *hs*βADR1 model shows that the average score of all amino acid residues is 0.16 which is relatively closed to 0.20. Moreover, ERRAT, a protein structure verification web server was used to verify the model on the basis of model building and refinement, and is extremely useful in making decisions about reliability of the homology model. ERRAT results showed that the overall quality factor for the *hs*βADR1 model is 73.35%., suggesting that the generated model is robust and can be use for virtual screening purpose in future. The 3D model of *hs*βADR1 revealed an excellent agreement with the experimentally determined 3D structure of *Mg*βADR1. (Fig 4) shows the superimposed view of *hs*βADR1 model and *Mg*βADR1 structure. The calculated polypeptide backbone (Cα, C and N atoms) root mean square deviation (RMSD) of *hs*βADR1 model against *Mg*βADR1 is 0.13 Ǻ. This low RMSD value indicates the resemblance of the modeled polypeptide backbone with the template. However, RMSD values slightly vary at C-terminus due to the sub-model region (209–254) of the target protein. Additionally, the modeled *hs*βADR1 is also superimposed on PDB IDs: 2YCW, 2YCX, 2YCY, and 2YCZ with RMSD values of 0.873 Ǻ, 0.973 Ǻ, 0.894 Ǻ, and 0.871 Ǻ, respectively (S5 Fig). These PDBs have comparable sequence similarities, identities and source as that of the template but some conformational changes has been observed for helix6 [36]. However, we found no observable conformational changes especially for those amino acid residues that are involved in molecular interactions with high-affinity antagonists I32, P32 and CAU located within H6 and CL-3. Finally, the *hs*βADR1 model is subjected to the sequence manipulation suite Ident and Sim [37] to observe the similarity and identity of the model with respect to the template structure. The results are better but afterwards much improved after manual editing of the target and template sequences, similarity and identity ratios are increased from 73% to 75.4% and 67% to 68.4%, respectively.

### 3D structural details of *hs*βADR1 homology model

The overall topology and secondary structural elements particular for the class A GPCR family remain quite conserved in the model of *hs*βADR1, that is, an extracellular N-terminus domain, seven 7-TM domains linked by three intracellular cytoplasmic loops (CL-1, CL-2 and CL-3), three extracellular loops (EL-1, EL-2 and EL-3), and a cytoplasmic C-terminus domain. The N-terminus domain comprises of nine amino acids residues (1–9) that are located outside the membrane. The TM-1–TM-7 helices spans from 10–34, 44–67, 80–104, 125–146, 198–173, 297–274 and 308–326 amino acid residues, respectively, while the C-terminus domain comprises of amino acid residues ranging from 327 to 342 at the inner face of membrane. The cytoplasmic loops, (CL-1, CL-2 and CL-3) comprise of residues 35–43, 105–124 and 199–273, respectively. The cytoplasmic loops CL-2 and CL-3 are believed to be important in the binding, selectivity or specificity and activation of G-proteins [38]. The extracellular loops, (EL-1, EL-2 and EL-3) comprising 68–79, 147–172 and 298–307 residues, respectively. Two conserved disulfide bridges which are important for cell surface expression, ligand binding, receptor activation and maintenance of the secondary structure are located in EL-2 and EL-3 regions at positions Cys81-Cys166 and Cys159-Cys165, respectively (Table 5).

**Table 5:** Structural description of template *Mg*βADR1 and modeled *hs*βADR1.

Structural description of *β*ADR1	Template *Mg*βADR1 (Number of Amino Acid Residues)	Modeled *hs*βADR1 (Number of Amino Acid Residues)
**N-terminus region**	1–9	1–9
**TM 1**	11–37	10–34
**CL-1**	38–46	35–43
**TM 2**	47–70	44–67
**EL-1**	71–82	68–79
**TM 3**	83–107	80–107
**CL-2**	108–128	108–124
**TM 4**	129–147	125–146
**EL-2**	148–172	147–172
**TM 5**	173–199	173–198
**CL-3**	200–233	199–273
**TM 6**	234–256	274–297
**EL-3**	257–265	298–307
**TM 7**	266–284	308–326
**C-terminus**	285–315	327–342
**Disulfide bridges**	Cys114-Cys119, Cys192-Cys198	Cys81-Cys166, Cys159-Cys165

### Conserved motifs of *hs*βADR1 homology model

DRY motif also known as ionic lock switch [39] is observed at position Asp105(3.49), Arg106(3.50) and Tyr107(3.51) in helix 3 of *hs*βADR1 model. The conserved Asp in DRY motif at the cytoplasmic end of helix 3 believes to regulate the transition state of active state, while the adjacent Arg is crucial for G-protein activation [40]. Another conserved penta-peptide NPXXY motif known as Tyrosine toggle switch (where X usually represents a hydrophobic residue and N is rarely exchanged against D) located at the C terminus of TM-7 which contributes to GPCR internalization and signal transduction. Several site-directed mutagenesis studies revealed the importance of this motif in signaling [41]. The NPXXY motif is present at position Arg323(7.49), Pro324(7.50), Ileu325(7.51), Ileu326(7.52) and Tyr327(7.53) in the *hs*βADR1model. The direct interaction of NPXXY motif with helix 8 is likely to be very significant in regulating the interactions of the C-terminal end of the GPCRs with various other cellular components involved in signaling (e.g, the PDZ domain), sequestration, and internalization of GPCRs. The tyrosine residue of NPXXY motif plays a decisive role in the phosphorylation of the receptor, presumably by controlling the affinity and activation capacity for the cognate G-protein [42]. Conserved regions of LAXXD motif which is involved in ligand binding and receptor cycling present in TM-2 at position Lys50(2.46), Ala51(2.47), Ser52(2.48), Ala53(2.49), Asp54(2.50) [43]. In general, PDZ domains bind to a short region of the C-terminus of other specific proteins. These short regions bind to the PDZ domain by beta-sheet augmentation. This means that the beta sheet in the PDZ domain is extended by the addition of a further beta-strand from the tail of the binding partner protein. GMGL, Gly10(1.34), Met11(1.35), Gly12(1.36) and Val13(1.37), is the PDZ-binding motif located within the C-terminal domain of modeled *hs*βADR1 [44]. These domains help anchor TM to the cytoskeleton and hold together signaling complexes. PDZ domain have many functions, from regulating the trafficking and targeting of proteins to assembling signaling complexes, and networks designed for efficient and specific signal transduction [45]. The amphipathic amino acid residues present in helix 8 are conserved among all human GPCRs (residues 327–341), located between the TM7 bound with helix 7. The palmitoylation occurs at N-terminus while the biosynthesis of receptor and the proper regulation of surface expression occur at C-terminus of *hs*βADR1. The side chain of two crucial residues of helix8, Asp332(8.49) and Arg334(8.51), are projected within the hydrophilic interface involved in stimulatory G-protein (G_s_) activation while the residue Phe333(8.50) and Phe337(8.54) are buried in the hydrophobic core of the helix [46].

### Role of salt bridging in *hs*βADR1

Salt bridges play important roles in protein structure and function. Disruption and the introduction of a salt bridge reduce and increase the stability of the protein, respectively [47]. In membrane proteins, one expects salt bridges to be particularly important because of a smaller dehydration penalty (loss of favorable contacts with water) on salt bridge formation [48]. Charged groups become largely dehydrated when inserted into membranes, and therefore, experience a smaller change in hydration between non-salt-bridging and salt-bridging states. There should also be a smaller effect because of solvent screening, strengthening salt-bridge interactions [48]. Multiple salt bridges are observed in the homology model of *hs*βADR1; Asp154:Arg157, Asp209:Arg213, Asp332:Lys335, Glu155:Arg158, Glu200:Lys203 and Glu212:Arg213. In addition, salt bridges can also serve as key interactions in much the same way as disulfide bonds (S6 Fig).

### Benchmarking docking programs for *hs*βADR1 homology model

#### Re-docking analysis

**Fig 5:**
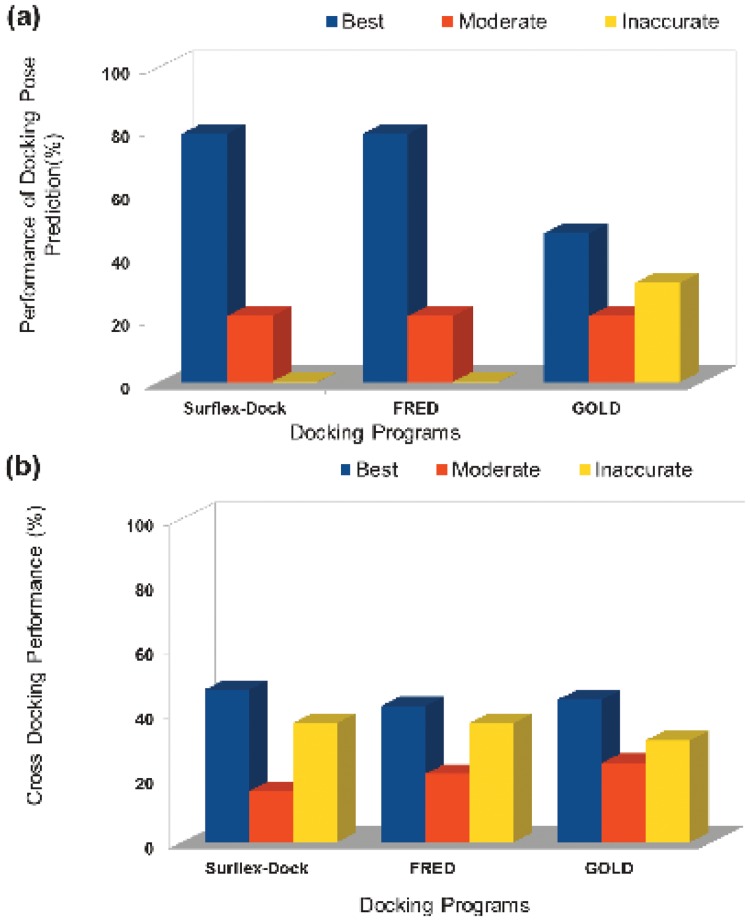
Graphical representation of re-docking and cross-docking results (A) re-docking, and (B) cross-docking results obtained by three docking software, Surflex-Dock, FRED and GOLD. Best, Moderate and Inaccurate results are shown by blue, red and yellow bars, respectively.

The success of docking is usually scrutinized by its accurate pose prediction ability[49–50], hence prior to the docking of βADR1agonists into the homology model of *hs*βADR1, the reliability of three docking programs including Surflex-Dock, FRED, and GOLD was assessed. The re-docking results were quantified on the basis of RMSD between the top-ranked docked conformation and the co-crystallized (termed as ˈreferenceˈ) ligand and visual analysis. The prediction is termed as Good when RMSD >1 or < 2 Å and the docked pose is superimposed on the ligand’s co-crystallized position, Fair when RMSD > 2 and < 3 Å and the docked pose is in active site but not superimposed on its native conformation, and Poor or Inaccurate when RMSD >3 Å and the docked pose is inverted or far away from the active site. The re-docking results showed that GOLD outperformed Surflex-Dock and FRED (Fig 5). Among the 19 complexes used, GOLD, Surflex-Dock, and FRED generated 100%, 74%, and 68% Good solutions in the top-ranked position, respectively. Surflex-Dock and FRED identified 5% and 10% Fair poses, respectively in the top-ranked docked poses. While both the programs generated 21% Inaccurate solutions in the top-ranked docked pose. The results are summarized in (Table 6).

**Table 6:** RMSDs obtained after re-docking analysis of 19 GPCRs complexes.

S. No.	PDB ID^†^	RMSDs (Å)		
		Surflex-Dock	FRED	GOLD*
1	1GZM_A	1.29	1.23	0.39
2	1GZM_B	1.09	1.33	0.40
3	1HZX_A	1.22	5.56	0.90
4	1HZX_B	1.68	5.55	0.68
5	2RH1_A	6.18	0.63	0.46
6	2VT4_A	0.78	0.76	0.74
7	2VT4_B	0.64	0.70	0.30
8	2Y00_A	1.07	0.81	0.72
9	2Y00_B	0.68	0.60	0.63
10	2Y01_A	1.04	1.03	0.18
11	2Y01_B	1.63	1.26	0.38
12	2Y02_A	7.00	7.71	0.43
13	2Y02_B	0.97	7.84	0.35
14	2Y03_A	2.03	0.75	0.36
15	2Y03_B	6.24	0.75	0.35
16	2Y04_A	1.22	0.50	0.40
17	2Y04_B	1.32	0.87	0.21
18	3D4S_A	3.14	2.42	0.14
19	3NY8_A	0.59	2.38	0.53

*RMSDs are reported on the consensus scoring functions in each case.

^†^PDB_ID_A/B represents the respective chain of homodimer PDB used for docking experiment.

### Cross-docking analysis

Furthermore, docking methods utilized in cross-docking is illustrated in (Table 7), was conducted to find out which program utilized in correctly ranks 19 ligands into their cognate binding site. The prediction was quantified on the basis of ligand's ranking (S2, S3 and S4) Tables. The cross-docking results indicates that Surflex-Dock is superior with 47% Best results in ranking the ligand in top 1–3 position in their cognate receptors. GOLD and FRED are returned with 42% and 44% best results, respectively (Fig 5). The position and the interaction of each ligand within the cognate receptor are visually analyzed. The results showed that the conformation of each ligand generated by Surflex-Dock is much better than the docked conformations generated by GOLD and FRED. Most of the interactions generated by Surflex-Dock are similar to the interactions present in the X-ray conformation. Hence, Surflex-Dock was found to be more appropriate for the docking of GPCR's ligands and it is further used in this study to explore the binding mode of *hs*βADR1 agonists into the active site of *hs*βADR1 model.

**Table 7:** Docking Method implemented in the study.

S. No.	Docking Method	Docking Runs	Scoring Function
**1**.	**Open Eyes Fred**	50 Runs	ShapeGauss, PLP, ChemGauss2, ChemGauss3, Chemscore, OEChemScore, ScreenScore, CGO, CGT and Consensus Score
**2**.	**GOLD**	50 Runs	Gold Score and ChemScore
**3**.	**Surflex-Dock**	50 Runs	Surflex- Score

### Analysis of binding modes of four agonists in the active site of *hs*βADR1 homology model

**Fig 6:**
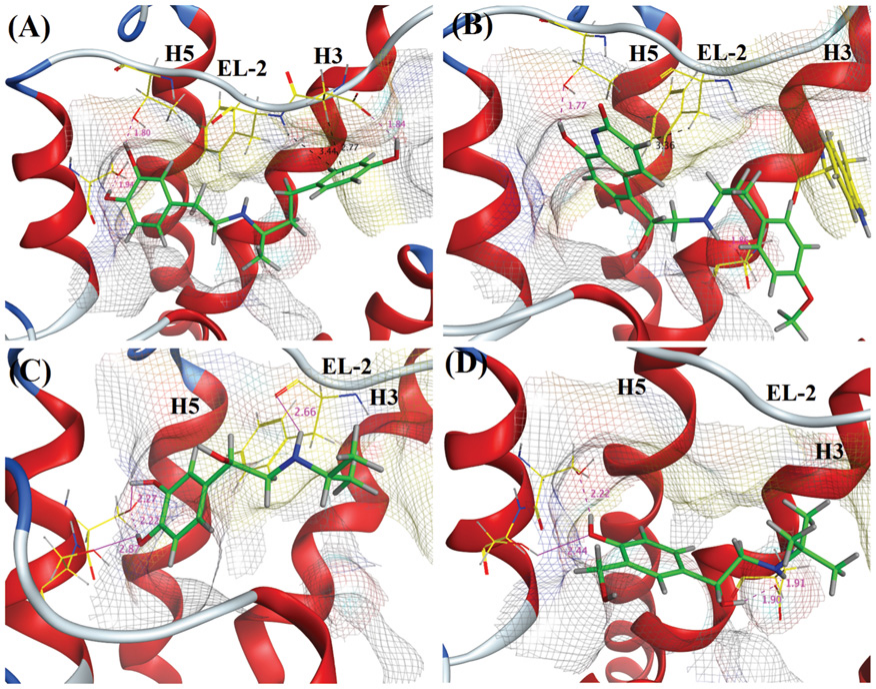
Binding modes of four agonists (A) Y00, (B) WHJ, (C) 5FW, and (D) 68H. Hydrogen bonding interactions are shown as magenta dashed lines. The most significant interactions are shown as magenta straight line. Cation-π interactions are represented as black color dashed lines (see also Table 9 and S7 Fig).

Based on re-docking and cross-docking performance, Surflex-Dock was used to predict the binding mode of *hs*βADR1 agonists into the ligand binding site of *hs*βADR1 model. For this purpose, four ligands namely Carmoterol (WHJ), Dobutamine (Y00), Isoprenaline (5FW) and Salbutamol (68H) were selected on th. The structures of these ligands are shown in S1 Fig. The binding modes of all four agonists revealed that they accommodate in the catecholamine binding pocket with similar orientation. The binding modes of agonists are depicted in Fig 6, S7 Fig, and Table 8 and Table 9.

**Table 8:** Four docked agonists along with their biological activities and interacted amino acid residues.

S. No.	Ligand ID	pEC_50_ Value (μM)	Surflex Score	Interacted Amino Acid Residues
1	Dobutamine (Y00)	-6.24±0.04	4.73	Trp84,Asp167,Phe168,Thr170, Ser178
2	Carmoterol (WHJ)	-8.37±0.10	3.07	Trp84,Asp88,Val89,Phe168,Thr170
3	Isoprenaline (5FW)	-7.86±0.10	3.67	Phe168,Ser178,Ser179
4	Salbutamol (68H)	-5.25±0.04	4.52	Asp88,Ser178,Ser179

**Table 9:** Specific location of interacted amino acid residues within the secondary structure of modeled *hs*βADR1 and the type of interactions involved with agonists.

Amino acid residue	Secondary Structure	Dobutamine (Y00)	Carmoterol (WHJ)	Isoprenaline (5FW)	Salbutamol (68H)
**Asp88**	H3	-	H-bond	-	H-bond^†^
**Asp167**	EL-2	H-bond, Cation-π	-	-	-
**Phe168**	EL-2	Cation-π	Cation-π	H-bond	-
**Thr170**	EL-2	H-bond	H-bond*	-	-
**Ser178**	H5	H-bond	-	H-bond^†^	H-bond
**Ser179**	H5	-	-	H-bond	H-bond

*shows three hydrogen bonding interactions with ligand.

^†^shows two hydrogen bonding interactions with ligand.

### Binding mode of Y00, WHJ, 5FW, 68H

The docked pose of Y00 reveals that multiple hydrogen bonding interactions are formed between the surrounding amino acid residues that stabilize Y00 in the catechol binding pocket. The −OH group at the phenol moiety is involved in hydrogen bonding with the γ carboxylate side chain of Asp167 (1.83 Å). The substituted −OH group at *meta* and *para* positions of ring B shows hydrogen bonding interactions with the side chains γ −OH of Thr170 (1.93 Å) and Ser178 (1.80 Å), respectively. Furthermore, the side chain phenyl ring of Phe168 and the carboxylate of Asp168 provide cation-π stacking interactions to the phenolic moiety of Y00 that further helps to stabilize the orientation of agonist. (Fig 6A) displays the binding mode of compound Y00.

The binding mode of **WHJ** demonstrates that the amino group of **WHJ** mediates hydrogen bond with the side chain carboxylate of Asp88 at a distance of 1.86 Ǻ. Similarly, Thr170 γ –OH group probes hydrogen bonding interactions with multiple ligand atoms including N atom and O atom at a distance of 2.03 Ǻ and 2.64 Ǻ, respectively. The same Thr170 is also involved in forming hydrogen bond at a distance of 1.76 Ǻ, the most significant hydrogen bonding interaction for WHJ. Phe168 forms cation-π interaction with one of the fused aromatic ring of WHJ. The binding orientation of compound WHJ is shown in (Fig 6B).

The binding mode of 5FW shows that the *para* −OH moiety of 5FW establishes hydrogen bonding interaction with the side chain carboxylate of Ser179 at a distance of 2.87 Ǻ. Additionally; Ser178 forms bi-dentate hydrogen bonding with the *para* and *meta* −OH groups at distances of 2.22 Ǻ and 2.17 Ǻ, respectively. The main chain carbonyl moiety of Phe168 mediate hydrogen bond with the amino group of 5FW (2.66 Ǻ). The docked binding mode of compound 5FW is depicted in (Fig 6C).

As revealed in (Fig 6D), the –OH of 68H shows similar interactions as observed for compound 5FW. The *para* substituted −OH group of 68H mediates bi-dentate hydrogen bonds with the side chain −OH groups of Ser178 and Ser179 at distances of 2.22 Ǻ and 2.44 Å, respectively. Furthermore, Asp88 mediates bi-dentate interaction with the linear chain amino and −OH groups of 68H at a distance of 1.91 Ǻ and 1.90 Ǻ, respectively.

The binding mode analysis of agonists Y00, 5FW, 68H displays that the Ser178 plays crucial role in stabilizing the agonists within the catechol binding pocket of *hs*βADR1 homology model. The docking results reveals that Ser178 and Phe168 are crucial residues in ligand binding by providing H-bonding, and π- π interactions, respectively, thus helps in the activation of *hs*βADR1.

We intend to incorporate molecular dynamic simulation studies in order to investigate the dynamic behavior of protein-inhibitor complex formation in the near future; and the role of most important residues will be determined. The study will be helpful to pursue structure based drug design of *hs*βADR1 blockers.

## Conclusions

Human βADR1 is found to be involved in several cardiovascular diseases. The lack of crystal structure of *hs*βADR1 provoked us to apply *in silico* techniques to initiate the drug discovery process for *hs*βADR1. Hence, to understand the characteristics structural features of *hs*βADR1 and to execute the structure-based drug design strategy for *hs*βADR1, threading-based homology modeling of mammalian origin were applied in this study. The model possesses acceptable structural profiles. Furthermore, the binding modes of four *hs*βADR1 agonist were determined *via* molecular docking simulation. H-3, H-5, and EL-2 regions were found to be important in ligand binding. Several residues including Trp84, Asp88, Val89, Asp167, Phe168, Thr170, Ser178, and Ser179 are involved in direct interactions with the ligand. Among all, Ser178, and Phe168 provides H-bonding, π- π interactions, respectively, hence found to be crucial residue in ligand binding and for the activation of *hs*βADR1. We are also investigating the dynamic behaviour of the Apo and ligand bound forms of *hs*βADR1 that will be published in future.

*Note*: The coordinate file of *hs*ADRB1 is submitted to the publicly accessible Protein Model Database (PMDB) [51]; www.caspur.it/PMDB). The PMDB ID of *hs*ADRB1 is PM0079652 respectively.

## Supporting Information

S1 Fig 2D representation of the bound ligands of 11 GPCRs complexes utilized in this study (see also Table 2). (TIFF)

S2 Fig Multiple sequence alignment by ClustalW. **(A)** Raw multiple sequence alignment, **(B)** manually edited multiple sequence alignment, and **(C)** manually edited multiple sequence alignment of template and target only. High conservation quality is found for micro domains, such as LAxxD motif in TM2, D/ERY motif in TM3, NpxxY motif in TM7, helix 8 and the position of the disulfide bond between Cys81 and Cys166 of EL-2 and Cys159 and Cys165 near the extracellular end of TM3 loop.
(TIF)

S3 Fig 3D representation of 5 sub-models generated by I-TASSER. **(A)** Sub-model 1, **(B)** Sub-model 2, **(C)** Sub-model 3, **(D)** Sub-model 4, and **(E)** Sub-model 5.
(TIF)

S4 Fig Ramachandran plot of modeled hsβADR1. (TIF)

S5 Fig Superimposed structure of modeled *hs*βADR1 (golden ribbon) with (A)2YCW, (B)2YCX, (C)2YCY, and (D)2YCZ. (TIF)

S6 Fig Multiple salt bridges as observed in the homology model of *hs*βADR1. **(A)** Asp154:Arg157, **(B)** Asp209:Arg213, **(C)** Asp332:Lys335, **(D)** Glu155:Arg158, **(E)** Glu200:Lys203, and **(F)** Glu212:Arg213.
(TIF)

S7 Fig 2D depiction of molecular interactions observed for all four ligands, (A)Y00,(B)WHJ, (C) 5FW, and (D) 68H within the active site of the homology model of *hs*βADR1. Only the most significant hydrogen bonding interactions are shown (see also Table 8 and (Fig 6)).
(TIF)

S1 Table Alignment scores (A) Alignment scores obtained from the alignment scores Raw Multiple Sequence Alignment (B) Alignment scores obtained from the Manually Edited Multiple Sequence Alignment (C) Alignment scores obtained from the Raw Target and Template Pair wise Sequence Alignment. (DOC)

S2 Table Cross-docking results of Surflex-Dock analyzed the basis of ranking of the cognate ligand in their respective receptor. Criteria for ranking: 1–3 position is best (green cell), 4–5 is moderate (blue cell) and >5 is Inaccurate (red cell).
(DOC)

S3 Table Cross-docking results of FRED analyzed on the basis of ranking of the cognate ligand in their respective receptor. Criteria for ranking: 1–3 position is best, 4–5 is moderate and >5 is Inaccurate.
(DOC)

S4 Table Cross-docking results of GOLD analyzed on the basis of ranking of the cognate ligand in their respective receptor. (DOC)
