# Supplementary figures and images for: 3D Structure Prediction of Human β1-Adrenergic Receptor via Threading-Based Homology Modeling for Implications in Structure-Based Drug Designing
Source: PLoS One. 2015 Apr 10;10(4):e0122223. doi: 10.1371/journal.pone.0122223 (PMC4393300; doi:10.1371/journal.pone.0122223)

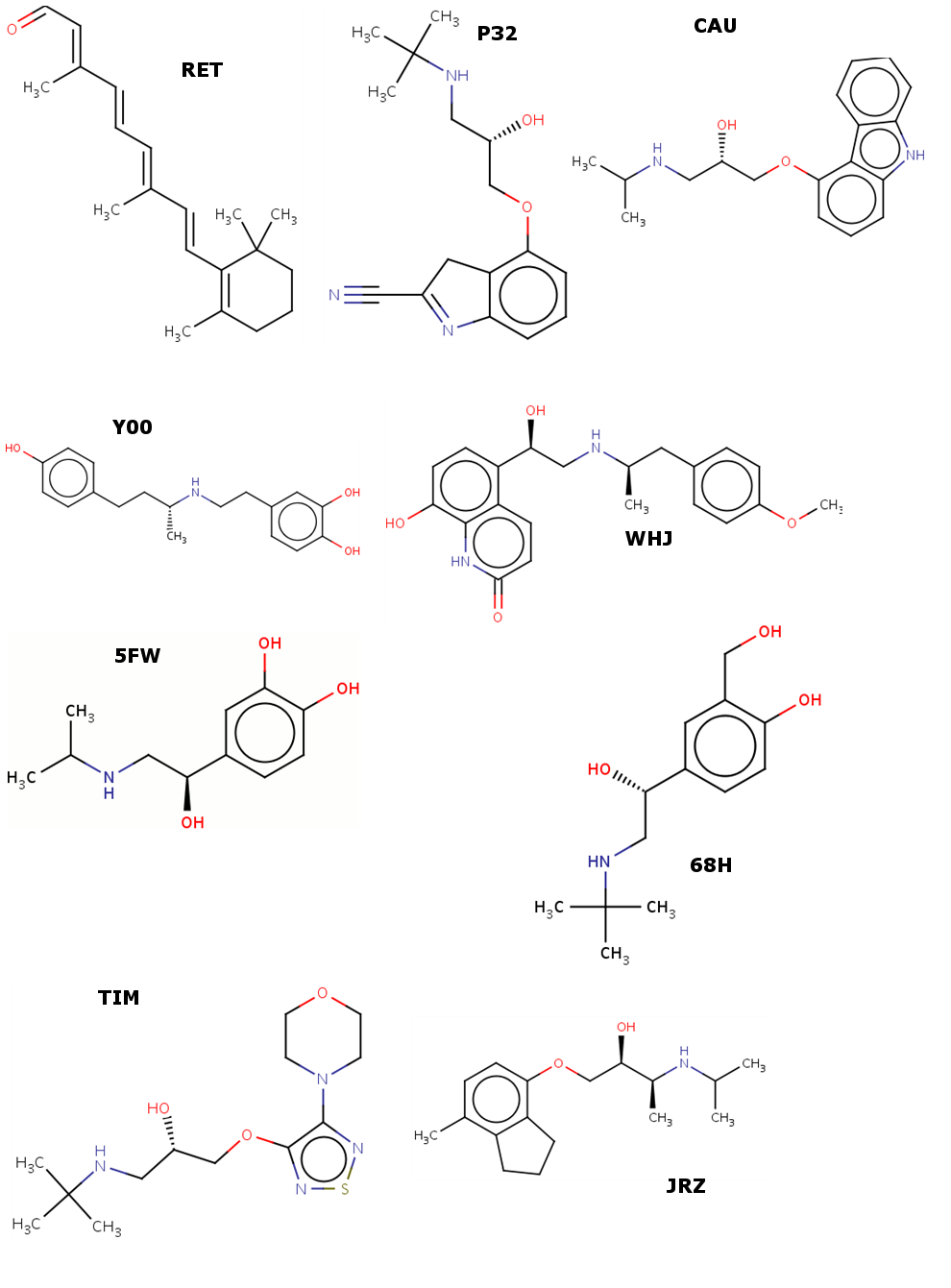

Supplement: S1 Fig — (TIFF) [file pone.0122223.s001.tiff]

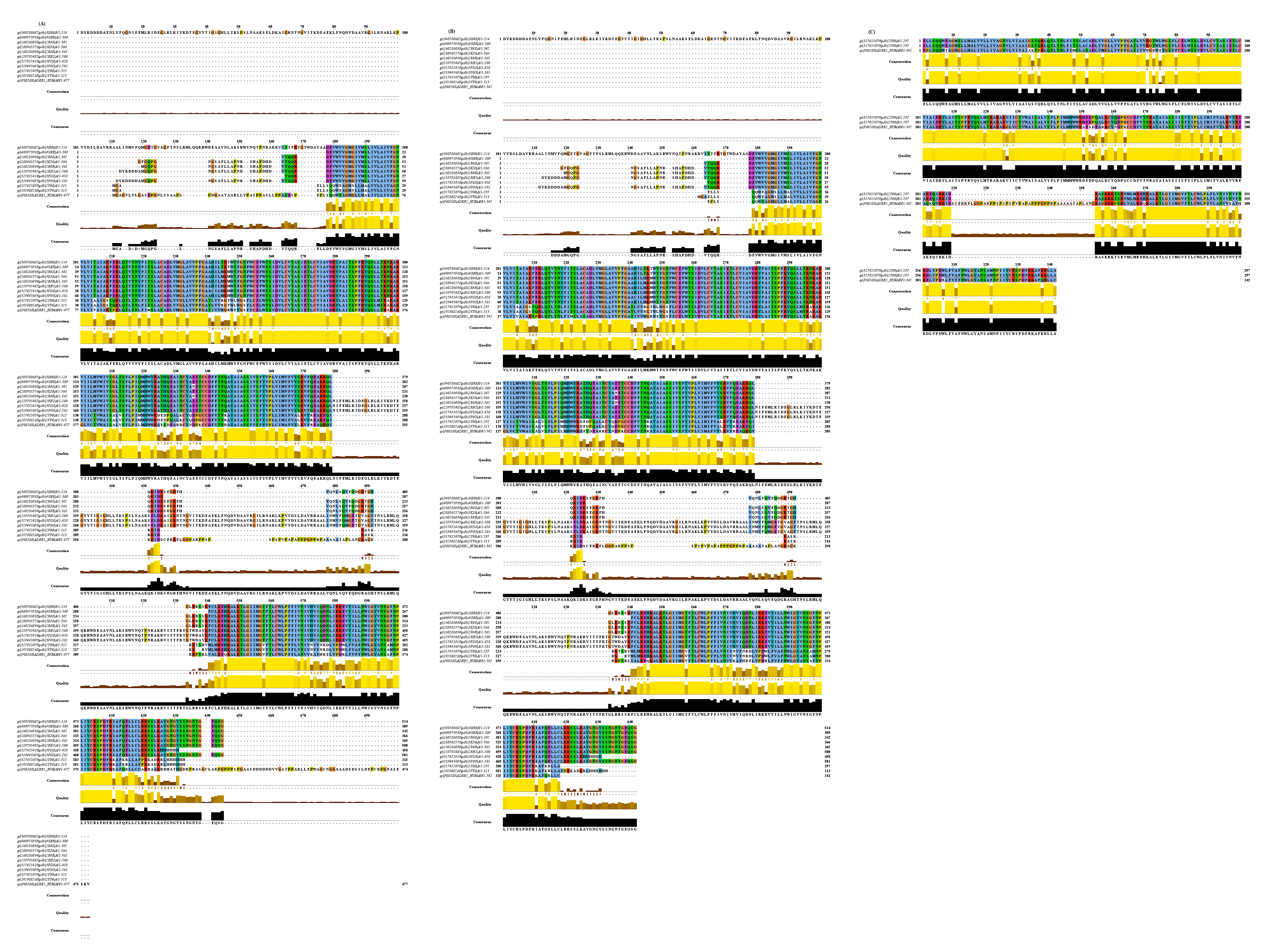

Supplement: S2 Fig — (A) Raw multiple sequence alignment, (B) manually edited multiple sequence alignment, and (C) manually edited multiple sequence alignment of template and target only. High conservation quality is found for micro domains, such as LAxxD motif in TM2, D/ERY motif in TM3, NpxxY motif in TM7, helix 8 and the position of the disulfide bond between Cys81 and Cys166 of EL-2 and Cys159 and Cys165 near the extracellular end of TM3 loop. (TIF) [file pone.0122223.s002.tif]

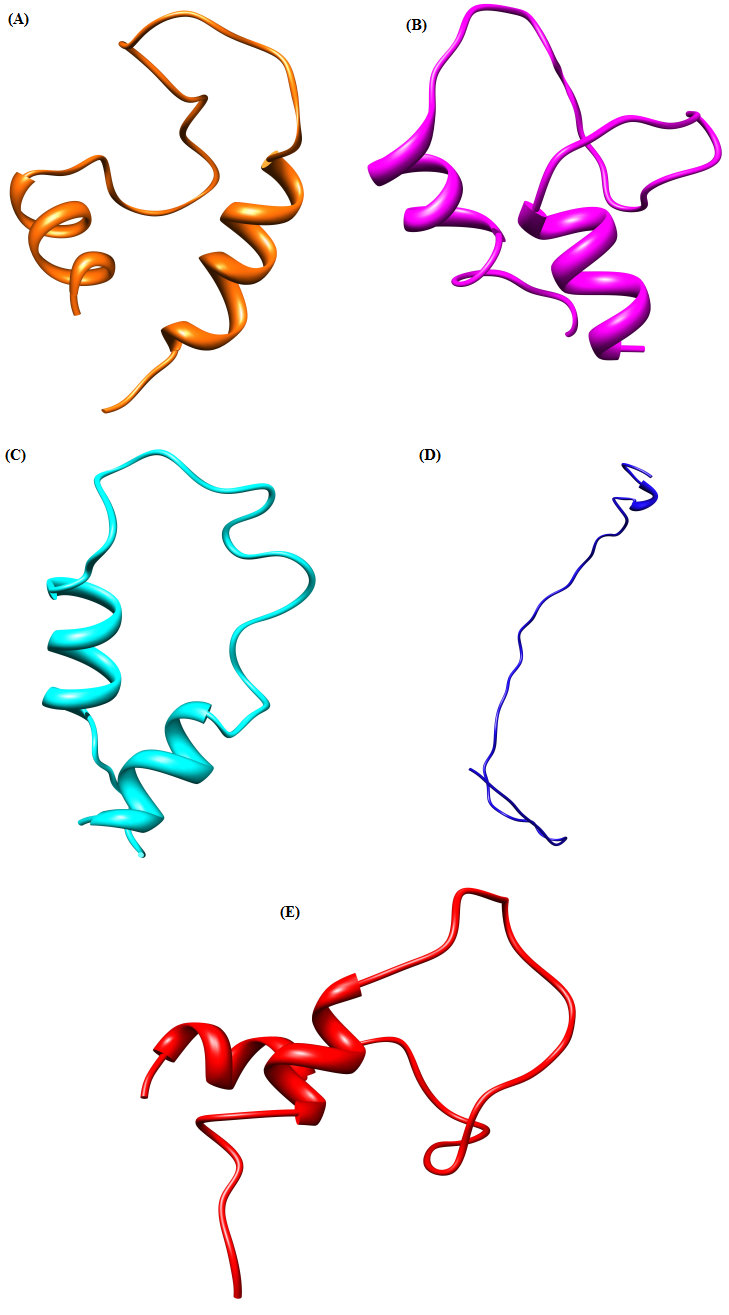

Supplement: S3 Fig — (A) Sub-model 1, (B) Sub-model 2, (C) Sub-model 3, (D) Sub-model 4, and (E) Sub-model 5. (TIF) [file pone.0122223.s003.tif]

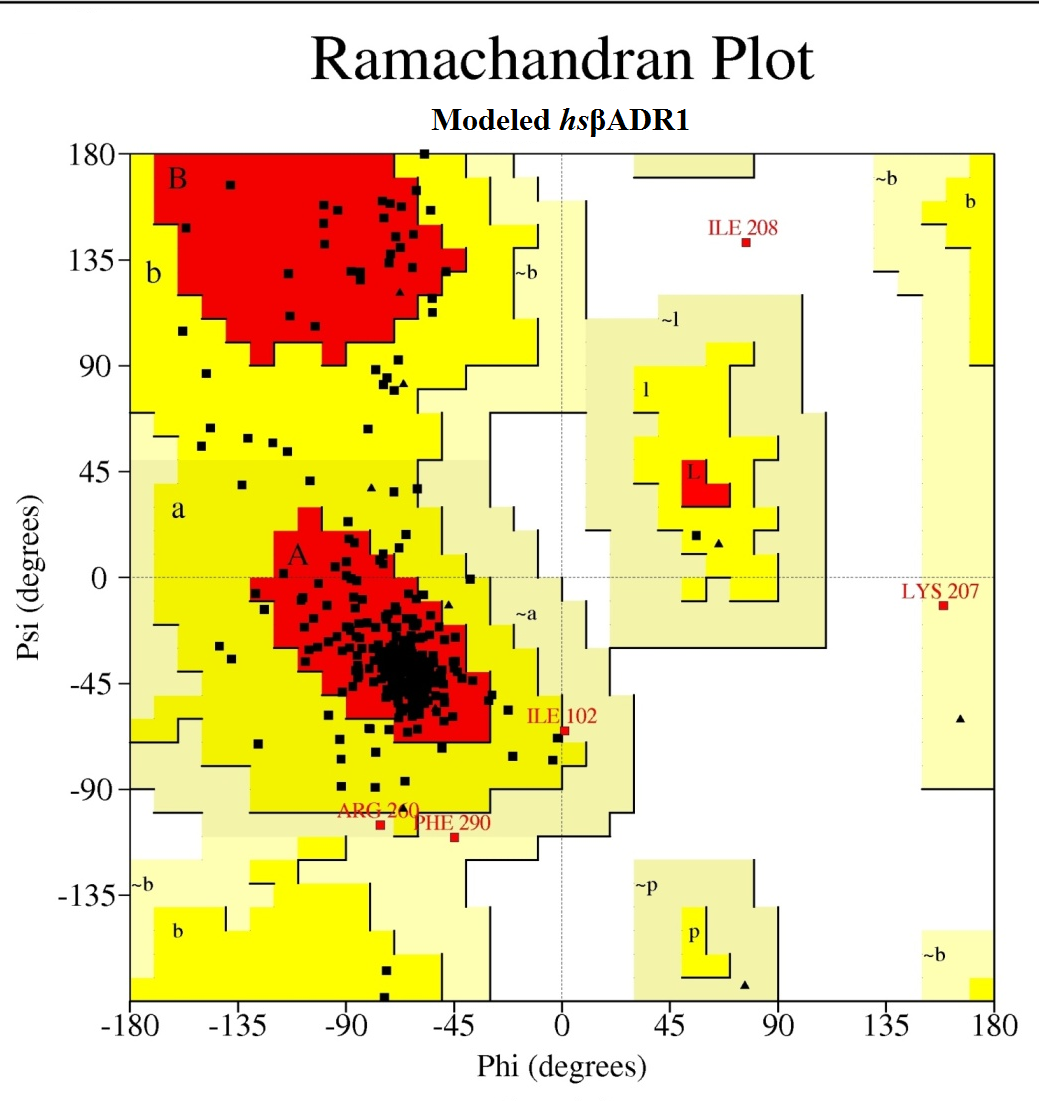

Supplement: S4 Fig — (TIF) [file pone.0122223.s004.tif]

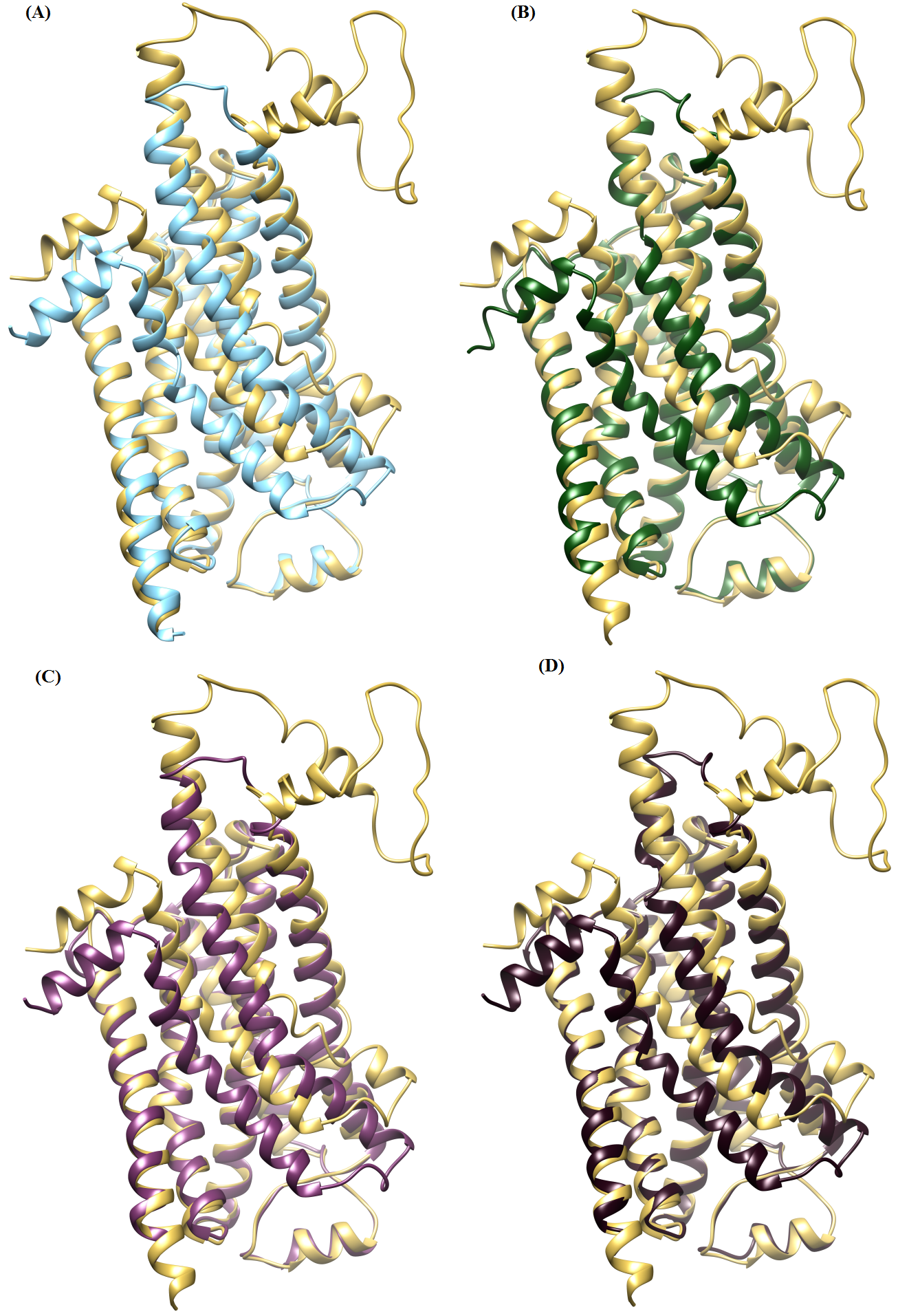

Supplement: S5 Fig — (TIF) [file pone.0122223.s005.tif]

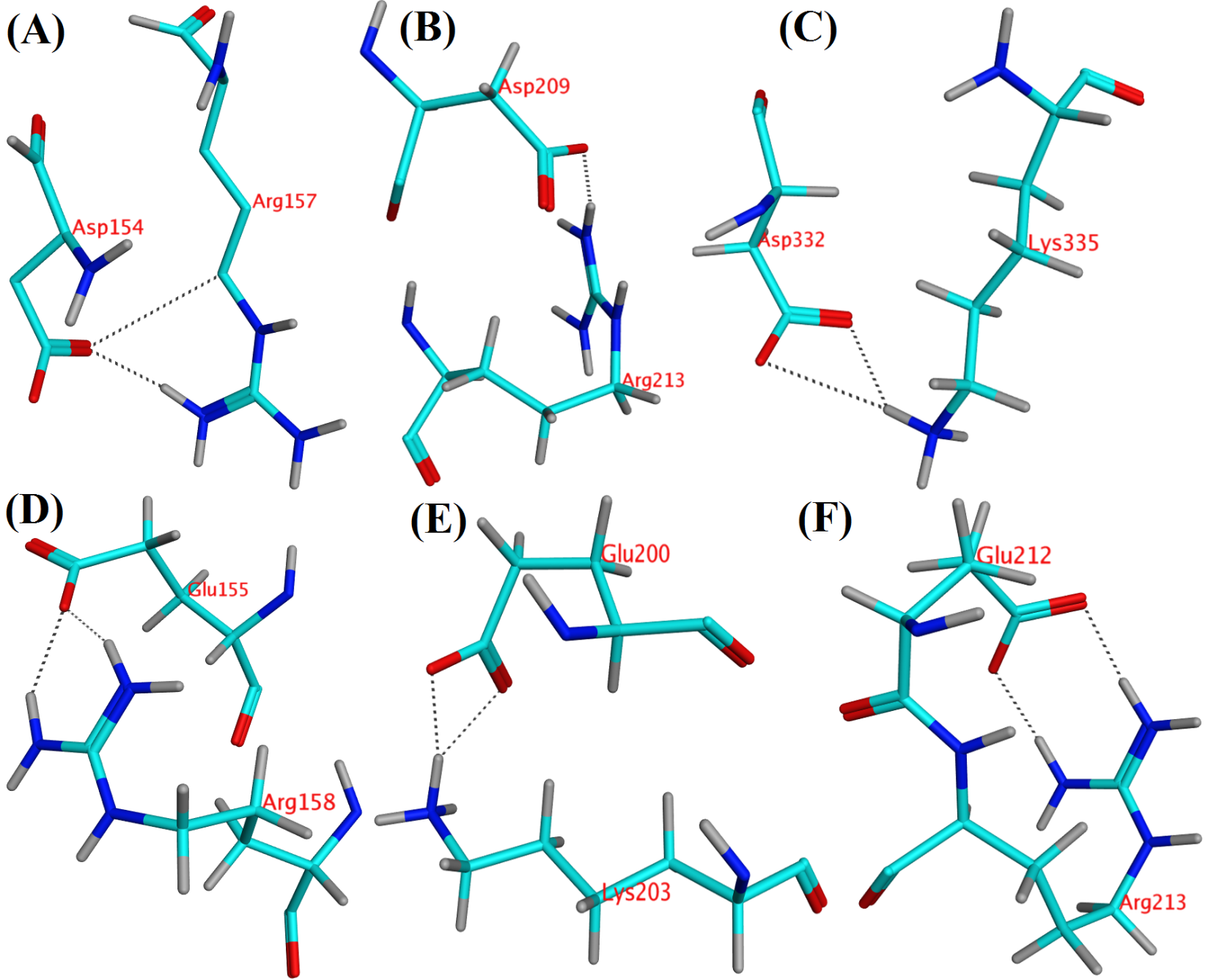

Supplement: S6 Fig — (A) Asp154:Arg157, (B) Asp209:Arg213, (C) Asp332:Lys335, (D) Glu155:Arg158, (E) Glu200:Lys203, and (F) Glu212:Arg213. (TIF) [file pone.0122223.s006.tif]

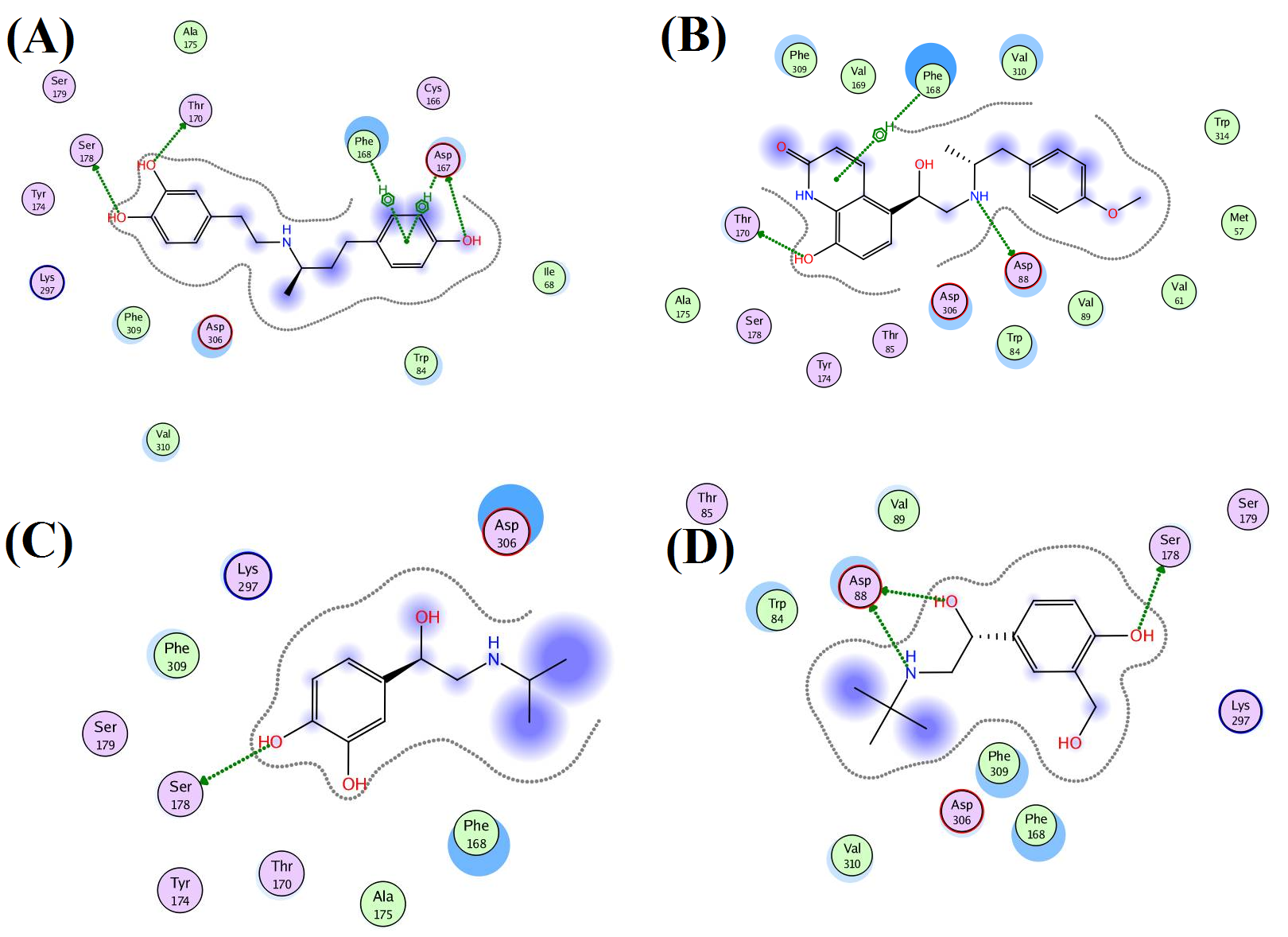

Supplement: S7 Fig — Only the most significant hydrogen bonding interactions are shown (see also Table 8 and (Fig 6)). (TIF) [file pone.0122223.s007.tif]
