# Supplementary material for: 3D Structure Prediction of Human β1-Adrenergic Receptor via Threading-Based Homology Modeling for Implications in Structure-Based Drug Designing
Source: PLoS One. 2015 Apr 10;10(4):e0122223. doi: 10.1371/journal.pone.0122223 (PMC4393300; doi:10.1371/journal.pone.0122223)
Supplement: S1 Table — (DOC) [file pone.0122223.s008.doc]

**Table S1A.** Alignment scores obtained from Raw Multiple Sequence Alignment.

| **Raw Multiple Sequence Alignment** | | | | | | | |
| --- | --- | --- | --- | --- | --- | --- | --- |
|
| **S. No.** | **Sequence A** | **Name** | **Length** | **Sequence B** | **Name** | **Length** | **Alignment Score** |
| **1** | 1 | P08588 | 477 | 2 | 2Y00 | 315 | 75.87 |
| **2** | 1 | P08588 | 477 | 3 | 2VT4 | 313 | 75.4 |
| **3** | 1 | P08588 | 477 | 4 | 2R4R | 365 | 53.42 |
| **4** | 1 | P08588 | 477 | 5 | 3KJ6 | 366 | 53.28 |
| **5** | 1 | P08588 | 477 | 6 | 2R4S | 342 | 56.43 |
| **6** | 1 | P08588 | 477 | 7 | 3SN6 | 514 | 40.46 |
| **7** | 1 | P08588 | 477 | 8 | 4GBR | 309 | 60.84 |
| **8** | 1 | P08588 | 477 | 9 | 3P0G | 501 | 40.46 |
| **9** | 1 | P08588 | 477 | 10 | 2RH1 | 500 | 40.46 |
| **10** | 1 | P08588 | 477 | 11 | 3PDS | 458 | 42.14 |
| **1** | 2 | 2Y00 | 315 | 3 | 2VT4 | 313 | 99.68 |
| **2** | 2 | 2Y00 | 315 | 4 | 2R4R | 365 | 58.1 |
| **3** | 2 | 2Y00 | 315 | 5 | 3KJ6 | 366 | 58.1 |
| **4** | 2 | 2Y00 | 315 | 6 | 2R4S | 342 | 58.1 |
| **5** | 2 | 2Y00 | 315 | 7 | 3SN6 | 514 | 58.41 |
| **6** | 2 | 2Y00 | 315 | 8 | 4GBR | 309 | 58.9 |
| **7** | 2 | 2Y00 | 315 | 9 | 3P0G | 501 | 56.83 |
| **8** | 2 | 2Y00 | 315 | 10 | 2RH1 | 500 | 56.83 |
| **9** | 2 | 2Y00 | 315 | 11 | 3PDS | 458 | 58.1 |
| 1 | 3 | 2VT4 | 313 | 4 | 2R4R | 365 | 58.47 |
| **2** | 3 | 2VT4 | 313 | 5 | 3KJ6 | 366 | 58.47 |
| **3** | 3 | 2VT4 | 313 | 6 | 2R4S | 342 | 58.47 |
| **4** | 3 | 2VT4 | 313 | 7 | 3SN6 | 514 | 58.79 |
| **5** | 3 | 2VT4 | 313 | 8 | 4GBR | 309 | 59.22 |
| **6** | 3 | 2VT4 | 313 | 9 | 3P0G | 501 | 57.51 |
| **7** | 3 | 2VT4 | 313 | 10 | 2RH1 | 500 | 57.51 |
| **8** | 3 | 2VT4 | 313 | 11 | 3PDS | 458 | 58.79 |
| **1** | 4 | 2R4R | 365 | 5 | 3KJ6 | 366 | 99.45 |
| **2** | 4 | 2R4R | 365 | 6 | 2R4S | 342 | 100 |
| **3** | 4 | 2R4R | 365 | 7 | 3SN6 | 514 | 91.51 |
| **4** | 4 | 2R4R | 365 | 8 | 4GBR | 309 | 99.03 |
| **5** | 4 | 2R4R | 365 | 9 | 3P0G | 501 | 92.33 |
| **6** | 4 | 2R4R | 365 | 10 | 2RH1 | 500 | 92.33 |
| **7** | 4 | 2R4R | 365 | 11 | 3PDS | 458 | 80.82 |
| **1** | 5 | 3KJ6 | 366 | 6 | 2R4S | 342 | 99.71 |
| **2** | 5 | 3KJ6 | 366 | 7 | 3SN6 | 514 | 91.53 |
| **3** | 5 | 3KJ6 | 366 | 8 | 4GBR | 309 | 99.35 |
| **4** | 5 | 3KJ6 | 366 | 9 | 3P0G | 501 | 92.08 |
| **5** | 5 | 3KJ6 | 366 | 10 | 2RH1 | 500 | 92.08 |
| **6** | 5 | 3KJ6 | 366 | 11 | 3PDS | 458 | 80.87 |
| **1** | 6 | 2R4S | 342 | 7 | 3SN6 | 514 | 97.66 |
| **2** | 6 | 2R4S | 342 | 8 | 4GBR | 309 | 99.03 |
| **3** | 6 | 2R4S | 342 | 9 | 3P0G | 501 | 91.81 |
| **4** | 6 | 2R4S | 342 | 10 | 2RH1 | 500 | 91.81 |
| **5** | 6 | 2R4S | 342 | 11 | 3PDS | 458 | 86.26 |
| **1** | 7 | 3SN6 | 514 | 8 | 4GBR | 309 | 100 |
| **2** | 7 | 3SN6 | 514 | 9 | 3P0G | 501 | 63.47 |
| **3** | 7 | 3SN6 | 514 | 10 | 2RH1 | 500 | 61 |
| **4** | 7 | 3SN6 | 514 | 11 | 3PDS | 458 | 63.32 |
| **1** | 8 | 4GBR | 309 | 9 | 3P0G | 501 | 98.06 |
| **2** | 8 | 4GBR | 309 | 10 | 2RH1 | 500 | 98.06 |
| **3** | 8 | 4GBR | 309 | 11 | 3PDS | 458 | 92.23 |
| **1** | 9 | 3P0G | 501 | 10 | 2RH1 | 500 | 99.4 |
| **2** | 9 | 3P0G | 501 | 11 | 3PDS | 458 | 98.47 |
| **1** | 10 | 2RH1 | 500 | 11 | 3PDS | 458 | 98.47 |

**Table S1B. Alignment scores obtained from Raw Manually Edited Multiple Sequence Alignment.**

| **Manually Edited Multiple Sequence Alignment** | | | | | | | |
| --- | --- | --- | --- | --- | --- | --- | --- |
|
| **S. No.** | **Sequence A** | **Name** | **Length** | **Sequence B** | **Name** | **Length** | **Alignment Score** |
| **1** | 1 | P08588 | 342 | 2 | 2Y00 | 297 | 78.45 |
| **2** | 1 | P08588 | 342 | 3 | 2VT4 | 313 | 73.48 |
| **3** | 1 | P08588 | 342 | 4 | 2R4R | 365 | 56.14 |
| **4** | 1 | P08588 | 342 | 5 | 3KJ6 | 366 | 56.14 |
| **5** | 1 | P08588 | 342 | 6 | 2R4S | 342 | 56.14 |
| **6** | 1 | P08588 | 342 | 7 | 3SN6 | 514 | 56.14 |
| **7** | 1 | P08588 | 342 | 8 | 4GBR | 309 | 60.52 |
| **8** | 1 | P08588 | 342 | 9 | 3P0G | 501 | 55.56 |
| **9** | 1 | P08588 | 342 | 10 | 2RH1 | 500 | 55.56 |
| **10** | 1 | P08588 | 342 | 11 | 3PDS | 458 | 55.56 |
| **1** | 2 | 2Y00 | 297 | 3 | 2VT4 | 313 | 98.99 |
| **2** | 2 | 2Y00 | 297 | 4 | 2R4R | 365 | 61.28 |
| **3** | 2 | 2Y00 | 297 | 5 | 3KJ6 | 366 | 61.28 |
| **4** | 2 | 2Y00 | 297 | 6 | 2R4S | 342 | 61.28 |
| **5** | 2 | 2Y00 | 297 | 7 | 3SN6 | 514 | 61.62 |
| **6** | 2 | 2Y00 | 297 | 8 | 4GBR | 309 | 60.94 |
| **7** | 2 | 2Y00 | 297 | 9 | 3P0G | 501 | 59.93 |
| **8** | 2 | 2Y00 | 297 | 10 | 2RH1 | 500 | 59.93 |
| **9** | 2 | 2Y00 | 297 | 11 | 3PDS | 458 | 59.93 |
| **1** | 3 | 2VT4 | 313 | 4 | 2R4R | 365 | 58.47 |
| **2** | 3 | 2VT4 | 313 | 5 | 3KJ6 | 366 | 58.47 |
| **3** | 3 | 2VT4 | 313 | 6 | 2R4S | 342 | 58.47 |
| **4** | 3 | 2VT4 | 313 | 7 | 3SN6 | 514 | 58.79 |
| **5** | 3 | 2VT4 | 313 | 8 | 4GBR | 309 | 59.22 |
| **6** | 3 | 2VT4 | 313 | 9 | 3P0G | 501 | 57.51 |
| **7** | 3 | 2VT4 | 313 | 10 | 2RH1 | 500 | 57.51 |
| **8** | 3 | 2VT4 | 313 | 11 | 3PDS | 458 | 58.79 |
| **1** | 4 | 2R4R | 365 | 5 | 3KJ6 | 366 | 99.45 |
| **2** | 4 | 2R4R | 365 | 6 | 2R4S | 342 | 100 |
| **3** | 4 | 2R4R | 365 | 7 | 3SN6 | 514 | 91.51 |
| **4** | 4 | 2R4R | 365 | 8 | 4GBR | 309 | 99.03 |
| **5** | 4 | 2R4R | 365 | 9 | 3P0G | 501 | 92.33 |
| **6** | 4 | 2R4R | 365 | 10 | 2RH1 | 500 | 92.33 |
| **7** | 4 | 2R4R | 365 | 11 | 3PDS | 458 | 80.82 |
| **1** | 5 | 3KJ6 | 366 | 6 | 2R4S | 342 | 99.71 |
| **2** | 5 | 3KJ6 | 366 | 7 | 3SN6 | 514 | 91.53 |
| **3** | 5 | 3KJ6 | 366 | 8 | 4GBR | 309 | 99.35 |
| **4** | 5 | 3KJ6 | 366 | 9 | 3P0G | 501 | 92.08 |
| **5** | 5 | 3KJ6 | 366 | 10 | 2RH1 | 500 | 92.08 |
| **6** | 5 | 3KJ6 | 366 | 11 | 3PDS | 458 | 80.87 |
| **1** | 6 | 2R4S | 342 | 7 | 3SN6 | 514 | 97.66 |
| **2** | 6 | 2R4S | 342 | 8 | 4GBR | 309 | 99.03 |
| **3** | 6 | 2R4S | 342 | 9 | 3P0G | 501 | 91.81 |
| **4** | 6 | 2R4S | 342 | 10 | 2RH1 | 500 | 91.81 |
| **5** | 6 | 2R4S | 342 | 11 | 3PDS | 458 | 86.26 |
| **1** | 7 | 3SN6 | 514 | 8 | 4GBR | 309 | 100 |
| **2** | 7 | 3SN6 | 514 | 9 | 3P0G | 501 | 63.47 |
| **3** | 7 | 3SN6 | 514 | 10 | 2RH1 | 500 | 61 |
| **4** | 7 | 3SN6 | 514 | 11 | 3PDS | 458 | 63.32 |
| **1** | 8 | 4GBR | 309 | 9 | 3P0G | 501 | 98.06 |
| **2** | 8 | 4GBR | 309 | 10 | 2RH1 | 500 | 98.06 |
| **3** | 8 | 4GBR | 309 | 11 | 3PDS | 458 | 92.23 |
| **1** | 9 | 3P0G | 501 | 10 | 2RH1 | 500 | 99.4 |
| **2** | 9 | 3P0G | 501 | 11 | 3PDS | 458 | 98.47 |
| **1** | 10 | 2RH1 | 500 | 11 | 3PDS | 458 | 98.47 |

**Table S1C. Alignment scores obtained from Raw Target and Template Pairwise Sequence Alignment.**

| **Raw Target and Template Pairwise Sequence Alignment** | | | | | | |
| --- | --- | --- | --- | --- | --- | --- |
| **Sequence A** | **Name** | **Length** | **Sequence B** | **Name** | **Length** | **Alignment Score** |
| 1 | P08588 | 477 | 2 | 2Y00 | 315 | 75.87 |
| **Manually Edited Target and Template Pairwise Sequence Alignment** | | | | | | |
| 1 | P08588 | 342 | 2 | 2Y00 | 297 | 78.45 |
