# Supplementary material for: 3D Structure Prediction of Human β1-Adrenergic Receptor via Threading-Based Homology Modeling for Implications in Structure-Based Drug Designing
Source: PLoS One. 2015 Apr 10;10(4):e0122223. doi: 10.1371/journal.pone.0122223 (PMC4393300; doi:10.1371/journal.pone.0122223)
Supplement: S3 Table — Criteria for ranking: 1–3 position is best, 4–5 is moderate and >5 is Inaccurate. (DOC) [file pone.0122223.s010.doc]

| **PDB ID** | **LIG ID** | **ShapeGauss** | **PLP** | **ChemGauss2** | **ChemGauss3** | **Chemscore** | **OEChemScore** | **ScreenScore** | **CGO** | **CGT** | **Consensus Score** |
| --- | --- | --- | --- | --- | --- | --- | --- | --- | --- | --- | --- |
| 1GZM_A | RET | **7** | **2** | **8** | **7** | **4** | **3** | **8** | **3** | **2** | **6** |
| 1GZM_B | RET | **3** | **3** | **5** | **5** | **1** | **1** | **4** | **3** | **2** | **1** |
| 1HZX_A | RET | **5** | **4** | **6** | **3** | **1** | **1** | **6** | **3** | **2** | **3** |
| 1HZX_B | RET | **2** | **2** | **3** | **4** | **2** | **2** | **2** | **2** | **1** | **1** |
| 2RH1_A | CAU | **2** | **1** | **1** | **1** | **1** | **1** | **1** | **1** | **1** | **1** |
| 2VT4_A | P32 | **3** | **3** | **3** | **3** | **4** | **2** | **3** | **1** | **1** | **2** |
| 2VT4_B | P32 | **2** | **3** | **2** | **2** | **3** | **5** | **2** | **1** | **1** | **2** |
| 2Y00_A | Y00 | --- | --- | --- | --- | --- | --- | --- | --- | --- | --- |
| 2Y00_B | Y00 | --- | --- | --- | --- | --- | --- | --- | --- | --- | --- |
| 2Y01_A | Y00 | **2** | **1** | **1** | **2** | **2** | **2** | **2** | **5** | **1** | **1** |
| 2Y01_B | Y00 | **2** | **1** | **2** | **1** | **1** | **1** | **1** | **1** | **1** | **1** |
| 2Y02_A | WHJ | **1** | **4** | **2** | **1** | **8** | **3** | **6** | **4** | **8** | **3** |
| 2Y02_B | WHJ | **1** | **1** | **1** | **1** | **6** | **3** | **1** | **1** | **1** | **1** |
| 2Y03_A | 5FW | **10** | **10** | **7** | **8** | **6** | **9** | **10** | **1** | **1** | **9** |
| 2Y03_B | 5FW | **7** | **5** | **5** | **4** | **6** | **7** | **6** | **2** | **2** | **5** |
| 2Y04_A | 68H | **9** | 8 | **5** | **9** | **8** | **8** | **9** | **1** | **1** | **6** |
| 2Y04_B | 68H | **6** | **2** | **4** | **4** | **3** | **6** | **7** | **2** | **1** | **4** |
| 3D4S_A | TIM | **5** | 6 | **6** | **6** | **10** | **8** | **4** | **2** | **2** | **6** |
| 3NY8_A | JRZ | **8** | **3** | **5** | **4** | **4** | **6** | **9** | **5** | **4** | **5** |

**Table S3.**  Cross-docking results of FRED analyzed on the basis of ranking of the cognate ligand in their respective receptor. Criteria for ranking: 1-3 position is best, 4-5 is moderate and >5 is Inaccurate.
